# Factor Analysis of the Classroom Assessment Scoring System Replicates the Three Domain Structure and Reveals no Support for the Bifactor Model in German Preschools

**DOI:** 10.3389/fpsyg.2018.01232

**Published:** 2018-07-18

**Authors:** Lilly-Marlen Bihler, Alexandru Agache, Katharina Kohl, Jessica A. Willard, Birgit Leyendecker

**Affiliations:** Department of Developmental Psychology, Ruhr-University Bochum, Bochum, Germany

**Keywords:** childcare quality, teacher–child interactions, teaching through interaction, construct validity, factorial validity

## Abstract

The quality of early childhood education and care (ECEC) is important for children’s development. One instrument that was developed to assess an aspect of ECEC quality is the Classroom Assessment Scoring System for pre-kindergarten children (CLASS Pre-K). We examined the factorial validity of the instrument using data from 177 German preschool classrooms. The three-factor *teaching through interaction* model (Hamre et al., 2013) was contrasted to a one-factor, a two-factor, and a bifactor model as proposed by Hamre et al. (2014). Our results indicated that the three-factor structure with the domains of *emotional support*, *classroom organization*, and *instructional support* fit the data best. The fit of the *teaching through interaction* model was satisfying after adding a cross-loading of the dimension language modeling on *emotional support*, and two correlated residuals. Evidence of convergent and discriminant validity are provided. In terms of factor structure and pattern score comparisons, the results were similar to previous United States and German studies. The discussion concerns the justifiability of the factor model revisions and draws directions for further research. We concluded that our study offers further evidence of the applicability of the CLASS Pre-K for the assessment of teacher–child interaction quality in the German context.

## Introduction

Throughout the last few years, there has been a noticeable shift in German preschools from primarily caretaking to providing an educationally stimulating environment. A stimulating learning environment is considered to be of great importance in both research and practice because of the accumulated evidence of a direct association of ECEC quality and children’s development (Mashburn et al., 2008; Curby et al., 2009). This study examines the internal structure of one instrument, the CLASS Pre-K that serves to assess one aspect of ECEC quality.

Even though there is a broad consensus that assessing and increasing ECEC quality is important in promoting children’s development, there is an ongoing debate about how to define ECEC quality (La Paro et al., 2012b; Working Group on Early Childhood Education and Care, 2014), and definitions vary between cultures (Sheridan et al., 2009). However, it is widely accepted that ECEC quality is a multidimensional construct, which comprises aspects of structural and process quality (Working Group on Early Childhood Education and Care, 2014; Slot et al., 2015). Structural quality represents the quality of preschools’ infrastructure, for example, equipment, child–teacher ratio, and teacher’s formal education. Process quality represents children’s daily socio-emotional, physical, and instructional experiences in interactions with peers, teachers, and materials (Howes et al., 2008; Slot et al., 2015). Although structural quality features are assumed to be predictive of interaction quality, evidence is still inconclusive (NICHD, 2002; Mashburn et al., 2008; Slot et al., 2015).

A number of approaches, including observations, self-reports, and checklists, have been developed to assess ECEC quality. Among observational instruments, some aim to measure global quality by observing a broad range of measures associated with structural and process quality [e.g., the revised Early Childhood Environment Rating Scale (ECERS-R); Harms and Clifford, 1980; Harms et al., 1998]. Others aim to measure specific quality aspects. There are several instruments that focus specifically on interaction quality, for example, the Caregiver Interaction Scale (CIS; Arnett, 1989) and the Early Childhood Classroom Observation Measure (ECCOM; Stipek and Byler, 2004). At the moment, one of the most widely used instruments to assess effective teacher–child interactions is the Classroom Assessment Scoring System for pre-kindergarten children (CLASS Pre-K; Pianta et al., 2015).

**FIGURE 1:**
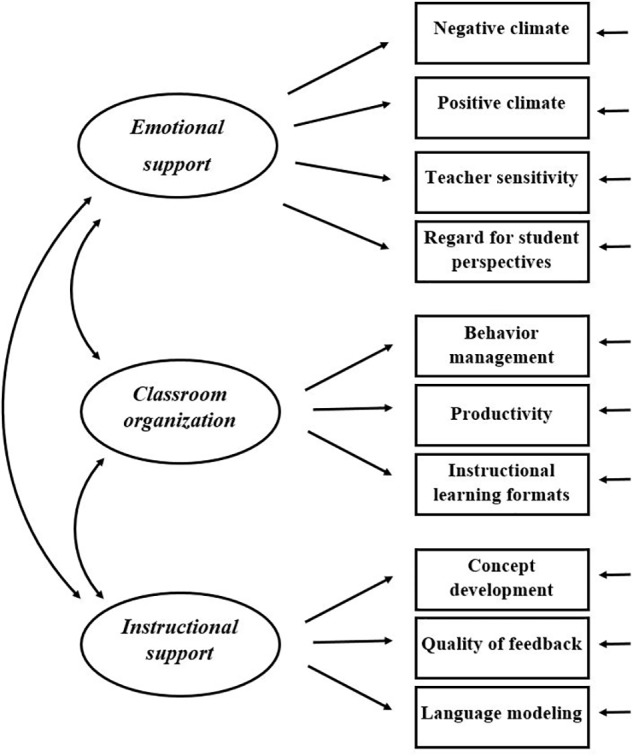
The three-factorial structure of the CLASS Pre-K as conceptualized by the *teaching through interaction* model (Pianta et al., 2015).

The CLASS Pre-K is an observational instrument designed to capture multiple dimensions of teacher–child interactions and to some degree, child–child interactions in preschool classrooms (Hamre et al., 2013; Pianta et al., 2015). The instrument has been shown to be a predictor of several child outcomes in preschool and in elementary school (Pianta et al., 2005; Mashburn et al., 2008). The conceptualization of the instrument builds on the theoretical framework of the *teaching through interaction* model (see **Figure 1**), which is based on Bronfenbrenner’s ecological system theory (Bronfenbrenner and Morris, 1998; Hamre et al., 2013). This theory posits that daily teacher–child interactions have a considerable effect on children’s learning. Pianta et al. (2015, p. 1) even state that teacher–child interactions are considered to be the “primary mechanism of student development and learning.”

The *teaching through interaction* model proposes three broad domains of interactions to be central to children’s positive development: emotional supportive interactions, teacher’s classroom management behaviors, and instructive interactions. Moreover, the model includes ten dimensions, each serving as an indicator for one of the three domains (Hamre et al., 2010, 2014). The first domain, e*motional support*, is based on attachment, self-determination, and motivation theory (Hamre et al., 2013). Attachment theory posits that children who feel comfortable, understood, and supported are likely to develop more positive social-emotional skills and to become more confident and autonomous (Bowlby, 1969). Self-determination and motivation theories propose that when children’s needs to feel autonomous, and competent are met, they will explore more and will be more motivated to learn (Elliot and Dweck, 2005; Ryan and Deci, 2008). The development of the second domain, *classroom organization*, is based on evidence that well-organized classrooms help children in developing self-regulating behavior and executive functioning skills, which are positively associated with children’s academic success (Morrison et al., 2010). The last domain, *instructional support*, was developed based on learning theories, e.g., on evidence that children learn more when teachers help them to gain a deeper understanding instead of providing rote instruction (Mayer, 2002).

**FIGURE 2:**
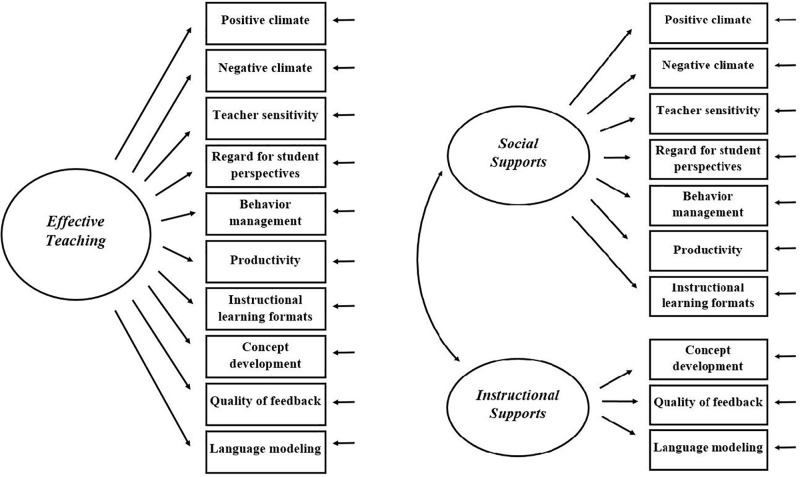
Alternative CLASS Pre-K one-factor **(left)**, and two-factor model **(right)** (Hamre et al., 2013).

The CLASS Pre-K is used widely in the United States, where it was developed. For example, it is used as a tool for monitoring the Head Start program and as a component of the Quality Rating and Improvement System of several states (Office of Child Care, 2015; Office of Head Start, 2018). Internationally, the CLASS Pre-K is also increasingly applied, for example, in Chile (Leyva et al., 2015) in China (Hu et al., 2016), in Germany (von Suchodoletz et al., 2014; Stuck et al., 2016), in Finland (Pakarinen et al., 2010), in the Netherlands (Slot et al., 2015), and in Portugal (Cadima et al., 2010). Studies validating the factorial structure of the CLASS consistently concluded that the three-factor *teaching through interaction* model is favorable to other potential structures, namely a one-factor and a two-factor model (see **Figure 2**; Pakarinen et al., 2010; Hamre et al., 2013; von Suchodoletz et al., 2014; Stuck et al., 2016). However, there were several country-specific findings that raise the question of whether it is appropriate to use this instrument outside of the United States. The scoring of the CLASS dimensions and the dimensions’ indicators could be culturally bound (Pastori and Pagani, 2017). A behavior (e.g., eye contact) may be interpreted differently in other cultural contexts or may not occur in some cultures. For example, in a Finnish study (Pakarinen et al., 2010), the dimension negative climate had to be excluded because of poor discriminant validity while studies from the United States reported a better distinction (Hamre et al., 2010, 2014). This finding suggests that the dimension did not adequately differentiate interaction quality in Finnish kindergarten classrooms (Pakarinen et al., 2010). Another example, albeit an anecdotal one, is that our observers were surprised as they watched videos from classrooms in the United States as a part of the CLASS Pre-K training. The enthusiasm of the teachers in these videos (as an indicator of the dimension positive climate) appeared exaggerated to them. They were unsure how to rate and weight this indicator in the German context because such displays of enthusiasm are rare in German culture. These are only two examples that demonstrate the importance of examining the validity of the CLASS Pre-K in specific cultural contexts.

There were also more methodical problems related to the *teaching through interaction* model. The three-factor model demonstrated an inadequate fit in a number of studies even after *post hoc* modifications were made (Pakarinen et al., 2010; Hamre et al., 2013; von Suchodoletz et al., 2014). In the Finnish study, a negative variance of the dimension language modeling had to be fixed to zero. By definition, a variance cannot be negative. A negative variance may indicate that the specified model is not appropriate for the data, but there could be also many other causes for a negative variance, for example, skewed variables (the Finnish classrooms scored very low in the dimension negative climate). In addition, in several studies a varying number of correlated residuals had to be allowed (Pakarinen et al., 2010; Leyva et al., 2015; Stuck et al., 2016). Correlated residuals indicate that dimensions have a common cause that is not explained by any of the domains in the *teaching through interaction* model. Thus, there may be an unknown feature underlying teacher–child interactions. Further, several studies found very high correlations among the domain scores, in particular among *emotional support*, and *classroom organization* (range of *r*s = 0.75–0.98; Pakarinen et al., 2010; Hamre et al., 2013; von Suchodoletz et al., 2014; Stuck et al., 2016). If the domains *emotional support* and *classroom organization* correlate highly, this might indicate that a teacher characteristic, for example, teacher’s experience or teacher’s well-being, influences both domains. Another interpretation would be that the dimensions of the highly correlated domains represent only one single domain. For example, the two-factor model proposes that emotional supportive interactions and teacher’s classroom organization behaviors can be summarized as a single domain, *social supports*. Further, highly correlated domains implicate potential multicollinearity. Consequently, the individual importance of each domain in predicting an external variable is difficult to assess in linear regression models (Brown, 2015).

**FIGURE 3:**
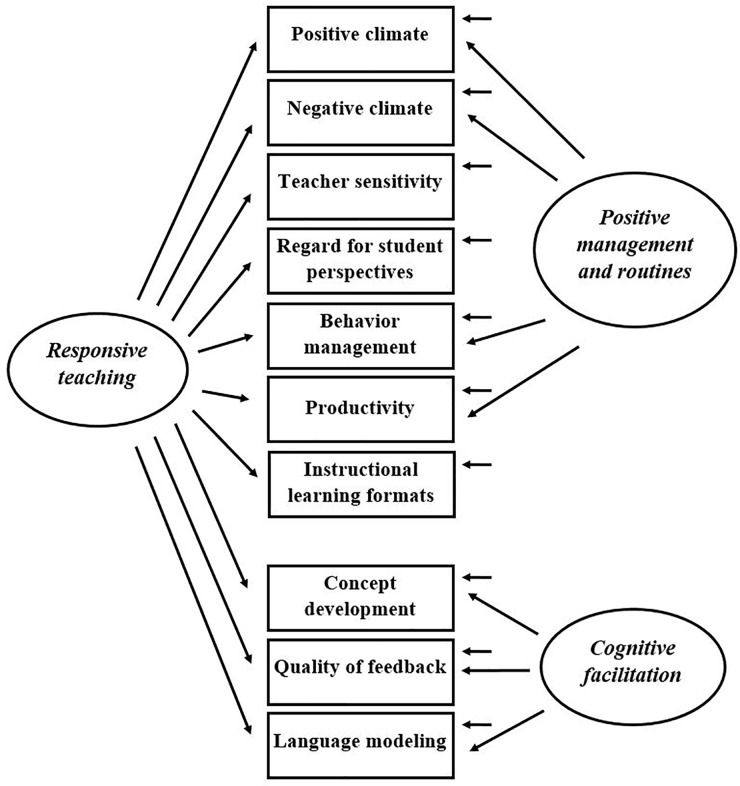
Best fitting bifactor model proposed by Hamre et al. (2014).

To address these issues, Hamre et al. (2014) proposed an alternative bifactor model that defines two specific domain-factors (*positive management and routines*, and *cognitive facilitation*) and a general domain-factor (*responsive teaching*, see **Figure 3**). The specific domains are conceptualized as interactions whose intent and content are aimed at fostering specific areas of children’s development. In contrast to the domains of the *teaching through interaction* model, the specific domains in the bifactor model are specified to be uncorrelated. Instead, the general domain is assumed to account for associations between domains by influencing all dimensions of the CLASS Pre-K. Thus, the bifactor model allows for the assessment of the individual importance of each specific domain in predicting child outcomes. All CLASS dimensions should load both on one specific domain and on the general domain. However, in the bifactor model of Hamre et al. (2014), three dimensions (teacher sensitivity, regard for student perspectives, and instructional learning formats) were constrained to load on the general domain only because their loading was non-significant.

High values in responsive teaching are assumed to be achieved by teachers who actively engage children in gaining classroom experiences, are consistently aware of and responsive to children’s cues, follow the lead of children by listening actively, respond accordingly to children’s statements, and ask follow-up questions to maintain exchanges (Hamre et al., 2014). Methodologically, the general responsivity domain might account for correlated residuals reported in several studies (Pakarinen et al., 2010; Leyva et al., 2015; Stuck et al., 2016). For example, the correlated residual between the dimensions positive climate and behavior management (Leyva et al., 2015) could be explained by the fact that a responsive teacher is more likely to immediately match children’s affect (an indicator of positive climate), and to anticipate problem behavior (an indicator of behavior management). In a sample of 325 CLASS Pre-K observations, all fit indices of the bifactor model of Hamre et al. (2014) were superior to those of the three-factor *teaching through interaction* model.

In two further United States studies, the bifactor model was contrasted to the *teaching through interaction* model (Sandilos and DiPerna, 2014; Sandilos et al., 2016) using observational data of American kindergarten and elementary school classrooms. Results of both studies indicated that a modified *teaching through interaction* model fit the data better than the bifactor model of Hamre et al. (2014). One explanation might be that the bifactor model is appropriate only when observing preschool classrooms (Sandilos et al., 2016). In preschool, teachers may be more nurturing and particularly responsive. In kindergarten or school, teachers may focus more on fostering children’s autonomy and independence. Responsive teaching may be not a central feature anymore. This distinction would make the general responsivity domain redundant.

To our knowledge, the only study testing the bifactor model with a non-United States sample is the analysis of Chinese preschool data by Hu et al. (2016). This study reports equivalent support for the original *teaching through interaction* model and a modified bifactor structure in terms of model fit. Overall, previous comparisons of the three domain structure to the bifactor model are largely inconclusive as to which model is preferable. To date, we are aware of no German study that has contrasted the recently proposed bifactor model (Hamre et al., 2014) to the more established *teaching through interaction* model. Previous German studies only compared the fit of the one-, two-, and three-factor models and concluded that the three factor structure is best supported by the data (von Suchodoletz et al., 2014; Stuck et al., 2016). Further, these studies were limited by small sample sizes (*N*s < 64).

There are similarities but also differences between the German and the American ECEC systems, which make it necessary to investigate the internal structure of the CLASS Pre-K in a larger German sample. These differences and similarities will be addressed now. For our study, we refer to German “Kindertagesstätten” [child daycare centers] as “preschools.” Similar to those in the United States, German public preschools are regulated at the state level and are state-subsidized. Costs of preschool attendance depend on parents’ income and are comparatively low. Enrollment rates between ages three and six are comparable to the United States (OECD, 2017). Since August 2013, all children between the age of three and the start of compulsory school education (usually at age six) have a legal right to attend preschool in Germany (Achtes Sozialgesetzbuch, 1990). It is a special feature of the German preschool system that many children attend mixed-age groups. Most caregivers in preschools are state-certified educators who have received highly standardized training (Statistisches Bundesamt, 2017), whereas the training of caregivers in the United States is more diverse (Early et al., 2007; Howes et al., 2008). In Germany, caregivers in preschools have usually received training that focused on working with preschool children. It differs from the training received by teachers working in schools. Compared to the United States, there are few highly structured activities involving all children of a group, and children spend a lot of time in free-play (König, 2009), which customarily takes places outside. As the German saying goes: “there is no such thing as bad weather, only the wrong clothes.” The explicit promotion of children’s academic skills usually does not happen until the last year before enrollment into the first grade. There is no mandatory core curriculum for preschools. With regard to these differences, particularly the self-perceived role of German teachers, an instrument such as the CLASS Pre-K should be carefully examined for its applicability in Germany.

In sum, there is diverging evidence regarding the underlying factorial structure of the CLASS Pre-K. Moreover, differences between the German and the American ECEC system could result in different internal structures. The present study addresses this issue by offering a comprehensive test of the established one-, two-, and three-factor models and of the bifactor model in a sample of 177 German preschool classrooms. We had three research questions. First, we tested whether the one-, the two-, or the three-factor *teaching through interaction* model fit the data best. Based on previous German studies assessing the internal structure of the CLASS Pre-K, we expected the *teaching through interaction* model to be supported best by our data. Second, we examined whether the bifactor or the *teaching through interaction* model fits the data better. Even on an international level, there is a lack of testing of the bifactor model. Therefore, we had no clear expectations which model would be supported by our data. Third, we investigated the reliability and the convergent and discriminant validity of the best-fitting model. Consistent and accurate results can only be obtained by instruments demonstrating high reliability and validity.

## Materials and Methods

We used data from an ongoing longitudinal study on language development in preschools located in North Rhine-Westphalia (NRW), the most populous federal state in Germany.

### Sample

Our sample consisted of 95 state-subsidized preschools. Two thirds of the preschools received additional financial funding to promote children’s language development. On average, these preschools did not differ from the remaining one third of preschools which did not receive additional funding on a wide range of administrative and socio-spatial economic data. This comparability was achieved by conducting a propensity score matching for sample selection. Additionally, our sample is representative of the more than 9600 state-funded preschools in NRW with regard to multiple administrative and socio-spatial covariates (e.g., gender distribution of children attending preschool, average share of monolingual German children attending preschool, average unemployment rate in the surrounding area of the preschool). The geographical distribution of the preschools in our sample within the federal state is also representative. Further details on the sample will be provided upon request.

### Participants

On average, preschools were attended by 68 children (range 36–122). Most preschools (84%) had a “closed concept” in which children spend most of their preschool time with the same teachers and children in a homeroom. Fifteen preschools (16%) had an “open concept” in which children are grouped into homerooms, but they are encouraged to choose from a variety of activities offered in other rooms as well. A total of 177 preschool classrooms participated in our study. Within these classrooms, the average class size was 21, and the average proportion of girls was 48%. At the time of the observation, the average children’s age was 54 months, with the youngest being 2 months old, and the oldest 83 months old. The CLASS Pre-K was developed to observe interactions between teachers and children of 3 to 5 years old. This age bracket did not apply to all children in our sample. Nevertheless, we considered it appropriate to use the CLASS Pre-K because the percentage of children younger than 3 years (11%) and of children older than 5 years (12%) was rather low. One third of the children spoke at least one other family language apart from German. On average, teachers in the observed classrooms were 42 years of age and had 16 years of professional experience in preschools.

### Measure

We used the CLASS Pre-K to assess process quality within the classrooms. The instrument measures ten dimensions of teacher–child interactions. According to the *teaching through interaction* framework, each dimension serves as an indicator for one of three distinct domains: (1) *Emotional support:* High values are achieved by teachers who provide comfort and assistance to their students, thereby providing a feeling of security in the classroom. Negative behaviors, like threatening the children and (peer) aggression are not, or are only rarely, present. Teachers are highly aware of and responsive to students’ emotional and cognitive needs and promote their autonomy (Pianta et al., 2015). (2) *Classroom organization*: High values are achieved by teachers who manage children’s behavior in an effective and timely manner. The classroom functions like a well-oiled machine, e.g., materials are prepared, and children do not spend time waiting. Children’s “thirst for knowledge” is successfully aroused and maintained (Pianta et al., 2015). (3) *Instructional support*: High values are achieved by teachers who promote student’s higher-order learning skills, use feedback to expand, deepen knowledge, and help children to learn more complex language skills (Pianta et al., 2015).

Live observations for this study were conducted by 11 officially certified CLASS Pre-K observers. Prior to data collection, all observers participated in a 2-day training and passed a reliability test with an average score of 84% reliability. To pass the reliability test, all observers had to watch and score five videos recorded in classrooms in the United States. On a seven point scale, 80% of the scores had to be within one point of the master codes, and at least two out of five scores for each dimension had to be within one point of the master codes.

We asked each teacher not to be influenced by our presence. In preschools with a closed concept, we observed two (82%) or three classrooms (2%). In preschools with an open concept only one observation was reasonable due to the mixed classroom structure (16%). For each classroom, four observation cycles were obtained within 1 day, primarily in the mornings, between February and October 2016. Studies evaluating the reliability of the CLASS Pre-K reported that scores are moderately to highly stable throughout the morning (Curby et al., 2010; von Suchodoletz et al., 2014; Stuck et al., 2016) but are not stable across seasons. For example, Buell et al. (2017) reported that scores differed significantly between fall and spring over the course of 3 years. Given that our data is cross-sectional, and most of our observations were conducted during the spring months (109 times, 62%), followed by during the summer months (41 times, 23%), during the winter months (18 times, 10%), and during the fall months (9 times, 5%), our sample does not permit further investigation of the measurement invariance and latent mean differences for different times of year (e.g., using multiple group factor analysis). An observation cycle of the CLASS Pre-K lasted 20 min on average. The instruments’ manual specifies that observers should watch and code all activities in the classroom, but that they should not code recreation time, including outdoor free time (Pianta et al., 2015). However, outdoor free time is very common in German preschools. Thus, we included inside and outside observations because this combination provides a more complete picture of the interactional quality in German preschools. In several classrooms, one or more teachers completed organizational tasks during our observations. If this was clearly communicated to the children, and if children’s needs were still met, this did not have a negative impact on the rating of the dimensions.

### Data Analyses

First, we investigated whether the CLASS Pre-K scores were stable across the cycles by considering the correlations between the means of cycles 1–4, and the means of cycle 1, cycles 1–2 and cycles 1–3, respectively. Temporal stability across cycles can be regarded as a reliability proxy. If the scores are not stable across cycles, interaction quality cannot be reliably assessed.

In a next step, we compared the *teaching through interaction* model to an alternative one-factor and an alternative two-factor model using confirmatory factor analyses. The one-factor model consisted of a general *effective teaching* domain capturing the variance of all CLASS dimensions. The two-factor model consisted of the *instructional support* domain and the domain *social supports* that results from collapsing the *emotional support* and *classroom organization* domains of the *teaching through interaction* model into one single domain. In a next step, we compared the *teaching through interaction* model to a bifactor model. The bifactor model was specified as being composed of one general and two domain-specific factors. All factors were specified to be uncorrelated. The general factor loaded on all indicators and controls for covariances between the indicators. The domain-specific factors loaded on selected indicators only and accounted for unique variance in the indicators within these factors. Analyses were conducted based on the means of the four observation cycles for each class.

Confirmatory factor analysis is based on the assumption of independent observations. Participating classes in this study were partially nested in preschools, so that the observations are not independent. This can lead to underestimated standard errors (Hox, 2010). Multilevel confirmatory factor analysis is a technique used to handle dependency in the data. However, due to small cluster size (on average 1.86 classrooms per preschool), this technique may lead to an inflation of cluster level parameters (Clarke, 2008). To adjust for clustering, standard errors were corrected using the Mplus “type = complex” command (Muthén and Muthén, 2017). We dichotomized the dimension negative climate, because variance was very limited (see descriptive “Results” section). Dichotomization is a good option if a variable is obviously not distributed normally (Muthén and Speckart, 1983). We did not split the variable based on our data, such as by a mean or median split, but instead by a theoretically meaningful point (negative behaviors were observed or not). To account for this categorical variable, we employed a weighted least squares estimator (WLSMV) across all models.

In all models, one loading in each factor was set to one to scale the latent variables, and all measurement errors were initially specified to be uncorrelated. For estimating the one-, two-, and three-factor models, all dimensions were initially specified to load on one-factor only, and all factors were allowed to correlate. For estimating the bifactor model, all factors were specified to be uncorrelated, and all dimensions were specified to load on both the general and one domain-specific factor (Brown, 2015). To test for local convergent validity, we calculated the dimensions’ reliabilities (squared factor loadings, *R*^2^), the factors’ reliabilities (ρ; Raykov, 2008), and the average extracted variances (AVE). To test for discriminant validity, we used the conservative Fornell–Larcker criterion, which assesses whether each factor shares more variance with its own dimensions than with the correlated factors (Fornell and Larcker, 1981). Values below 1 indicate discriminant validity. All analyses were conducted in Mplus Version 8.

### Model Evaluation

Model fit was evaluated by multiple fit indices, as they provide different information. The model χ^2^ along with its degrees of freedom (*df*) and its *p*-value were considered. Excellent model fit was indicated by a *p*-value of >0.05. Further, the Root Mean Square Error of Approximation (RMSEA), the Comparative Fit Index (CFI), and the Tucker-Lewis index (TLI) were considered. In accordance with Hu and Bentler (1999), good model fit was defined by the following criteria: RMSEA ≤ 0.06, CFI ≥ 0.95, and TLI ≥ 0.95. All of these fit indices assess the overall model fit. However, it is just as important to ensure that no local areas of severe misspecification are present (Kline, 2011; Brown, 2015). For this purpose, MIs were inspected. High modification indices indicate that freeing a fixed or constrained parameter can significantly improve model fit. This improvement could be made by specifying correlated errors between two indicators or by specifying an indicator cross-loading with a second factor, provided there is a substantial foundation for this modification. We first freed the parameter with the highest MI and proceeded step by step because freeing one single parameter can remedy several high MIs (Brown, 2015).

## Results

### Descriptive Statistics

The means and standard deviations for the CLASS Pre-K dimensions in our sample are provided in **Table 1**. The dimension negative climate was rated lowest and had the smallest standard deviation (*M* = 1.26, *SD* = 0.33). Thus, hardly any expressed negativity, such as yelling or threatening, was observed. For our confirmatory factor analyses, we dichotomized the dimension into “no expressed negativity was observed” (0; 56%) and “expressed negativity was observed” (1; 44%). All dimensions of the domain *emotional support* were rated more positively in our German sample than in American comparison studies (Hamre et al., 2013; Sandilos and DiPerna, 2014). The dimensions of the domains *classroom organization* and *instructional support* were rated in or near the range of the American ratings. Furthermore, all domain scores were rated in the range of other German studies (von Suchodoletz et al., 2014; Stuck et al., 2016).

**Table 1:** Descriptives of our sample, and comparison with American samples.

	German sample (*N* = 177)	U.S. sample (*N* = 458 to *N* = 4341; Hamre et al., 2013)	U.S. sample (*N* = 314; Hamre et al., 2014)
	*M (SD)*	*M (SD)*	*M (SD)*
Positive climate	6.04 (0.72)	5.10 (0.80)	5.22 (0.99)
Negative climate	1.26 (0.33)	1.40 (0.61)	1.34 (0.64)
Teacher sensitivity	5.42 (0.78)	4.75 (0.90)	4.43 (1.25)
Regard for student perspectives	5.08 (0.94)	4.26 (0.88)	4.11 (1.27)
Behavior management	5.69 (0.77)	5.26 (0.91)	5.60 (0.97)
Productivity	4.92 (0.99)	4.14 (1.02)	5.75 (0.79)
Instructional learning formats	3.77 (1.01)	4.82 (0.94)	3.80 (1.22)
Concept development	1.81 (0.73)	2.58 (1.02)	1.76 (0.76)
Quality of feedback	2.48 (0.90)	2.54 (1.13)	2.52 (1.00)
Language modeling	2.83 (0.83)	2.81 (0.93)	2.80 (1.02)


The correlations between the dimensions (see **Table 2**) ranged from *r* = –0.07 (between positive climate and quality of feedback) to *r* = 0.71 (between productivity and instructional learning formats). With regard to the three-factor model, we found moderate to high correlations for all dimensions within a factor and mostly low correlations for dimensions between factors. This provides the first evidence of convergent and discriminant validity of the *teaching through interaction* model. However, we also found several high inter-correlations between dimensions belonging to different factors. For instance, the correlation between positive climate and behavior management was *r* = 0.56.

**Table 2:** Correlations between the dimensions (*N* = 177).

	1	2	3	4	5	6	7	8	9
(1) Positive climate	–								
(2) Negative climate	0.38^∗^	–							
(3) Teacher sensitivity	0.68^∗^	0.31^∗^	–						
(4) Regard for student perspectives	0.47^∗^	0.25^∗^	0.61^∗^	–					
(5) Behavior management	0.56^∗^	0.26^∗^	0.56^∗^	0.33^∗^	–				
(6) Productivity	0.32^∗^	0.22^∗^	0.46^∗^	0.42^∗^	0.54^∗^	–			
(7) Instructional learning formats	0.34^∗^	0.13	0.42^∗^	0.36^∗^	0.47^∗^	0.71^∗^	–		
(8) Concept development	0.03	0.16^∗^	0.15	0.12	-0.03	0.03	0.10	–	
(9) Quality of feedback	-0.07	0.16^∗^	0.22^∗^	0.19^∗^	0.01	0.27^∗^	0.22^∗^	0.49^∗^	–
(10) Language modeling	0.29^∗^	0.23^∗^	0.56^∗^	0.44	0.21^∗^	0.30^∗^	0.29^∗^	0.56^∗^	0.59^∗^


*Pearson correlations were calculated for pairs of continuous variables. Biserial correlations were calculated for pairs consisting of a dichotomous and a continuous variable. ^∗^p < 0.05.*

### Stability of Scores Across Cycles

We calculated correlations between the means of cycles 1–4 and the means of cycle 1, cycles 1–2 and cycles 1–3, respectively, to assess the stability of the CLASS Pre-K scores across cycles (see **Table 3**). All correlations were moderate to high, even those between scores of cycle 1 and the total score. This indicates that our CLASS scores were highly stable across the cycles and that four observation cycles were sufficient to assess classroom interactions.

**Table 3:** Stability of the scores across cycles (*n* = 177).

	Cycle 1 with total score	Cycles 1–2 with total score	Cycles 1–3 with total score
Positive climate	0.75	0.86	0.95
Negative climate^a^	0.59	0.78	0.92
Teacher sensitivity	0.67	0.83	0.94
Regard for student perspectives	0.73	0.86	0.95
Behavior management	0.74	0.86	0.94
Productivity	0.77	0.88	0.95
Instructional learning formats	0.69	0.84	0.95
Concept development	0.68	0.84	0.96
Quality of feedback	0.72	0.88	0.96
Language modeling	0.72	0.85	0.94
		


*Total score = mean of cycles 1–4. ^*a*^Dimension was not yet dichotomized*.

### Contrasting the *Teaching Through Interaction* Model to the One- and Two-Factor Models

We applied confirmatory factor analyses to compare the one-factor, two-factor, and *teaching through interaction* models. All analyses were conducted using the means of the four observation cycles per class. In the two- and three-factor models, negative residual variances for language modeling were estimated (improper solutions). In the two-factor model, this was remedied by specifying a cross-loading of language modeling on the *social supports* domain. In the three-factor model, two cross-loadings of language modeling on *emotional support* and *classroom organization* were specified with only the first being significant. Therefore, the model was re-specified with a single cross-loading of language modeling on *emotional support*. Although the revised three-factor model showed the best fit, none of the three models fit the data well according to the applied cut-off values. Therefore, the MIs were inspected, and the three-factor model was revised based on the highest MI of 15.26, which indicated correlated errors between positive climate and quality of feedback. In a next step, we also allowed correlated errors between positive climate and behavior management (MI = 13.84). Freeing these parameters led to satisfying model fit.

### Contrasting the *Teaching Through Interaction* Model to the Bifactor Model

In a next step, the bifactor model with two domain-specific factors from Hamre et al. (2014) was specified. A negative residual variance was estimated for the dimension behavior management (improper solution). We re-specified the model with the residual variance of behavior management fixed to zero and obtained a proper solution. Fixing the residual variance is tenable because it was non-significant. All dimensions demonstrated significant loadings on the domain-specific factors. However, the fit of the bifactor model was worse than the fit of the revised three-factor model (see **Table 4**).

**Table 4:** Fit indices for the tested models (*n* = 177).

	χ^2^	df	CFI	TLI	RMSEA
One-factor model	147.47^∗^	35	0.65	0.55	0.14
Two-factor model^a^	81.09^∗^	33	0.85	0.80	0.09
Three-factor model^b^	48.15^∗^	29	0.94	0.91	0.06
Bifactor model^c^	67.64^∗^	26	0.87	0.78	0.10


*^a^Revised by a cross-loading of language modeling on social supports. ^*b*^Revised by a cross-loading of language modeling on emotional support, correlated errors between positive climate and quality of feedback, as well as between positive climate and behavior management. ^c^Revised by fixing the residual variance of behavior management to zero. ^∗^p < 0.05.*

### Assessing Reliability, Convergent, and Discriminant Validity of the Final Three-Factor Model

**FIGURE 4:**
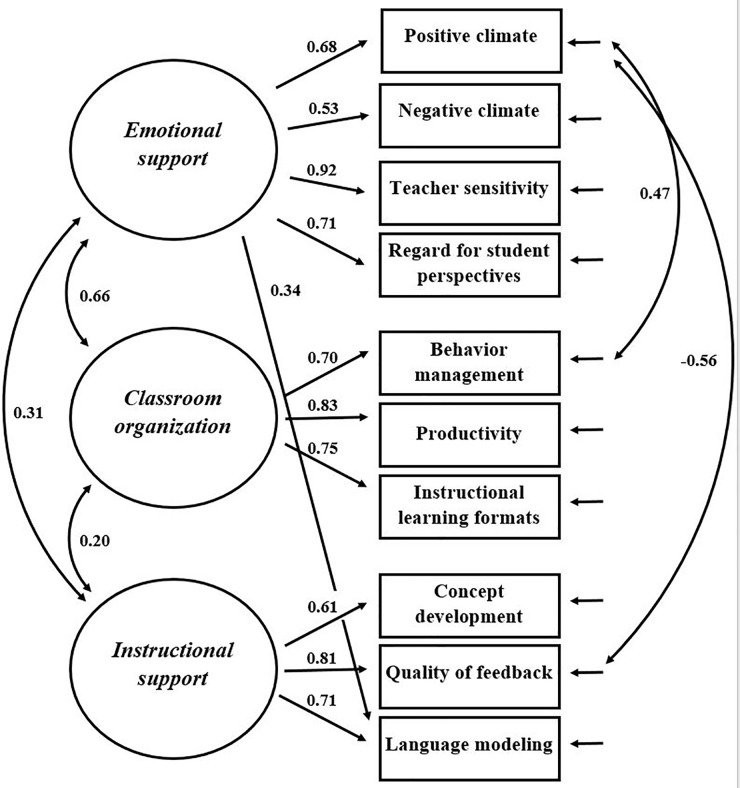
Revised three-factor model (a cross-loading of language modeling on *emotional support* and correlated errors between positive climate and quality of feedback, as well as between positive climate and behavior management were specified) with standardized parameter estimates.

**Figure 4** depicts the revised three-factor model with the standardized parameter estimates. The standardized factor loadings were all moderate to high, ranging from *r* = 0.53 (*emotional support* on negative climate) to *r* = 0.92 (*emotional support* on teacher sensitivity). Reliability estimates according to Cronbach’s alphas for the three-factors were α = 0.77 (*emotional support*), α = 0.80 (*classroom organization*), and α = 0.79 (*instructional support*). The reliabilities of the dimensions were moderate to high, ranging between *R*^2^ = 0.28–0.85 *(emotional support*), *R*^2^ = 0.48–0.68 (*classroom organization*) and *R*^2^ = 0.37–0.65 (*instructional support*). To assess factor reliability, the Raykov’s reliability coefficients were calculated. The coefficients were good, ranging from ρ = 0.79 (*instructional support*) to ρ = 0.83 (*emotional support*). The AVE was >0.50 for all three constructs. This supports the convergent validity of the factors.

With regard to discriminant validity, the factor correlations between *emotional support* and *instructional support* (*r*_s_ = 0.31) as well as between *classroom organization* and *instructional support* (*r*_s_ = 0.20) were low. In contrast, the factor correlation between *emotional support* and *classroom organization* was rather high with *r*_s_ = 0.66. However, the discriminant validity was supported according to the conservative Fornell–Larcker criterion. Each factor shared more variance with its own dimensions than with the correlated factors.

## Discussion

The CLASS Pre-K is increasingly used internationally to measure teacher–child interaction quality in ECEC systems. This widespread application makes it necessary to assess the reliability and validity of the instrument outside of the United States. Therefore, we investigated the internal structure of the CLASS Pre-K in a comparatively large sample of 177 German preschool classrooms. Contrasting the *teaching through interaction* model to a one-factor, a two-factor, and a bifactor model, our main finding was that the *teaching through interaction* model demonstrated the best fit for the data. Reliability and convergent and discriminant validity were also satisfying. Thus, the three-factor *teaching through interaction* model seems to be the most appropriate model to fit German preschool data. Surprisingly, we found no support for the bifactor model, which has been regarded as conceptually more appealing when compared to the three-factor model in the literature (Hamre et al., 2014). We discuss these results in the light of the German ECEC context.

### Contrasting the *Teaching Through Interaction* Model to the One-Factor and Two-Factor Models

Our results show that the three-factor *teaching through interaction* model fits the data better than a one- and a two-factor model. This outcome is consistent with findings of other studies conducted in Germany, Finland, and the United States (Pakarinen et al., 2010; Hamre et al., 2013; Stuck et al., 2016). Estimating the three-factor model resulted in a negative error variance for the dimension language modeling. Such an improper solution was also found by the study of Pakarinen et al. (2010). While the authors of the previous study adopted a rather technical solution to fix the error variance of language modeling to zero, we investigated the sources of this model behavior and found a significant cross-loading with the factor *emotional support*. What might explain this finding? Language modeling reflects not only teachers’ language-stimulating and language-facilitating techniques, but also children’s responsiveness to their teachers (Pianta et al., 2015). The cross-loading may be explained by the fact that children engage more in conversations in classrooms marked by greater *emotional support* because they feel comfortable, understood, and supported. Besides the cross-loading of the dimension language modeling with the factor *emotional support*, further model revision found two correlated errors, which may be due to method effects. An explanation for the negatively correlated error between positive climate and quality of feedback may be that teachers make fewer general positive comments on children’s efforts (a marker of positive climate) when concentrating on giving specific feedback that expands knowledge (a marker of quality of feedback). The second positively correlated error between positive climate and behavior management may be due to a more positive atmosphere in a classroom when behavioral expectations are clearly communicated by the teacher and met by the children and vice-versa. Overall, since the cross-loading and both correlated errors were small to moderate in size, these model revisions did not compromise the conceptual clarity of the three-factor model. The model demonstrated satisfying discriminant and convergent validity.

### Why Did the Bifactor Model Not Fit?

Hamre et al. (2014) reported a superiority of the bifactor model over the *teaching through interaction* model in a sample of American preschool classrooms. However, we could not replicate this finding in our sample of German preschool classrooms. One explanation might be that there is an overlap between what the general *responsive teaching* domain and what the *emotional support* domain represent. For example, teacher’s responsiveness is also an indicator of teacher’s sensitivity (a dimension of *emotional support*). Hence, if the general and the specific domains measure something similar, the decomposition into dimensions’ unique and shared variances is difficult. This feature could have caused the estimation problem which occurred in our study when initially specifying the bifactor model. Further, the model of Hamre et al. (2014) violates some of the basic bifactor modeling assumptions. In the model, three indicators did not load significantly on specific domains and were constrained to load on the general domain only (see **Figure 3**). These non-significant loadings point to conceptual caveats of the CLASS bifactor model *per se* because for these dimensions, there is no distinction between unique and shared variances (Eid et al., 2016).

### CLASS Quality Score Profiles in German Preschools

On a descriptive level, our CLASS mean domain scores are similar to those of previous German and international studies. Emotional supportive interactions were rated with the highest mean domain score, followed by teacher’s classroom management behaviors while instructive interactions were rated with the lowest mean domain score. For the practice, our results underscore the need for a continuous monitoring and improvement of the quality of instructional teacher–child interactions. It is noteworthy that all dimensions of the domain *emotional support* were rated comparatively higher in all German studies than in the American studies. This difference indicates that in Germany, teachers prioritize providing a caring environment. This focus is not surprising considering the central role of attachment theory in both German ECEC research and pedagogical praxis. Preschool teachers are trained to take on the role of the sensitive mother (Rosabal-Coto et al., 2017). Free play is qualified as a core activity, because children often decide what they want to do. For a better understanding of mean differences in CLASS scores, further research on the cross-cultural equivalence of CLASS ratings and of the definitions of high ECEC quality is needed. A study including behavioral markers on teacher–child interactions across samples from different countries would allow for the investigations of measurement invariance issues (e.g., do they have similar factor loadings?).

In line with previous studies carried out in Germany and Finland (Pakarinen et al., 2010; von Suchodoletz et al., 2014; Stuck et al., 2016), the dimension negative climate was consistently rated on the lower end of this scale and had a small standard deviation. It appears that a seven point scale may be too differentiated for the assessment of this dimension in German and Finnish preschools while studies from the United States usually reported a better distinction (Hamre et al., 2010, 2014). Dichotomizing the dimension into observed and not observed negative behaviors is one solution we applied. Another solution would be to make finer distinctions in the rating of the lower end of this scale.

### Practical Challenges in the Application of the CLASS Pre-K in German Preschools

The application of the CLASS Pre-K challenged us for several reasons. First, our presence seemed to influence the behavior of some teachers. However, over time, it no longer significantly affected them. Therefore, although our results show that score stability across cycles was satisfying for less than four cycles, we do not recommend reducing the number of cycles. A further challenge was that the CLASS Pre-K was developed for children ranging from 3 to 5 years of age. However, German preschools are also attended by children under 3 years and up to 6 years of age. For children younger than 3 years old, the CLASS Toddler is available (La Paro et al., 2012a). We decided against using this additional version because most of our observations were conducted in mixed-age classes (two CLASS versions would have had to be used) and because the number of children under 3 years old was comparatively low. In addition, the CLASS Toddler builds on a somewhat different theoretical framework than the CLASS Pre-K. For the CLASS Toddler, the authors propose a two-factor model composed of eight dimensions (La Paro et al., 2012a) rather than a three-factor model composed of ten dimensions. Even though the CLASS Pre-K and the CLASS Toddler differ in their dimensions and in their posited factor models, the revised *teaching through interaction* model fit our data well despite 10% of the children observed being younger than 3 years old. This finding indicates that using the CLASS Pre-K is appropriate even in countries such as Germany, where mixed-age classes in preschools are common. A further challenge was that we had uncertainties about the ratings of the indicators of two dimensions. As previously described, intense displays of enthusiasm are not typical of the German culture. Therefore, we did not place the same weight on this indicator as would have been placed in an American study. However, this did not seem to cause an underestimation of the positive climate score. On average, it was rated higher than in American samples. Furthermore, children in German preschools spend a lot of time in free-play. This time is usually neither well-structured nor strongly influenced by teacher-involved play (König, 2009). However, we still assigned high scores to the indicator clarity of learning objectives of the dimension instructional learning formats if there was clear evidence that children were aware that they could choose from a range of activities (even if a clear learning objective was not verbally communicated). Despite this rather liberal coding approach, the classrooms in our sample scored moderate on average on this dimension. Thus, the instrument also seems appropriate for ECEC systems in which children spend a lot of time in free-play.

### Limitations

Our sample of preschools is representative of those of the most populous federal state of Germany with regard to multiple administrative and socio-spatial covariates. This feature should be taken into account when generalizing to preschools in other German federal states.

This study did not investigate the predictive validity of the *teaching through interaction* model. Importantly, there is evidence that the domains do not have the same predictive validity for all children. For example, a recent study reported that instructive interactions were positively related to dual language learners’ German morphological skills, whereas they were not for monolingual German children (Bihler et al., 2018). Thus, appropriately investigating predictive validity is beyond the scope of this study. Future studies may address which additional characteristics moderate the relation between the CLASS Pre-K domains and child outcomes.

Another limitation concerns the factor analysis framework *per se*. Reflective factors models (including the bifactor approach) are special cases of the common cause factor model, which posits that the CLASS dimensions’ variances are solely explained by the latent variables. Our analyses also point to shared variances (i.e., cross-loading and the two residuals correlations) not captured by the latent variables. As shown above, these shared variances may occur in part due to shared method effects and may be specific only to our sample. Since the added cross-loading and the error covariances were small in size, it seems plausible that they do not compromise the conceptual clarity of the three-factor model. However, these shared variances may also be due to complex causal effects between the single CLASS domains (e.g., teacher sensitivity → language modeling → teacher sensitivity) that are not well captured by the factor analysis model. Further studies could investigate the nature of these shared variances by analyzing multiple CLASS ratings (e.g., multitrait-multimethod analyses) or by separating stable and occasional construct variances within longitudinal assessments (e.g., Latent State-Trait Models; Steyer et al., 1999).

## Conclusion

The purpose of this study was to examine the internal structure of the CLASS Pre-K in a comparatively large sample of German preschools. Our results indicate that a three-factor structure is preferable to alternative one, two, or bifactor solutions. The reliability and convergent and discriminant validity of the three-factor model are satisfying. Even though the application of the instrument presented some challenges that were mainly caused by specific features of the German culture, the instrument demonstrated to be applicable to the German context.

## Ethics Statement

This study was carried out in accordance with the recommendations for psychological research of the Deutsche Gesellschaft für Psychologie (DGPs; German Psychological Society). All parents and teachers received detailed written information prior to the start of the study. We did not collect any personal data. However, we obtained prior informed consent for other parts of the project, e.g., for completing questionnaires. All data was collected anonymously. The protocol was approved by the ethics committee of the Faculty of Psychology at the Ruhr University Bochum.

## Author Contributions

All authors designed the study and planned the data collection. L-MB conceived the original idea. AA and L-MB conducted the statistical analyses. L-MB drafted the manuscript. BL supervised the project. All authors were involved in writing the manuscript, discussing the results, and approving the submitted version.

## Conflict of Interest Statement

The authors declare that the research was conducted in the absence of any commercial or financial relationships that could be construed as a potential conflict of interest.
